# Comparative analysis of COVID‐19 case fatality rate between two waves in Nepal

**DOI:** 10.1111/irv.12922

**Published:** 2021-10-14

**Authors:** Sulochan GC, Ashok Khanal, Atmika Paudel, Vijay S. GC, Aashis Khanal, Suresh Panthee

**Affiliations:** ^1^ Institute of Medicine Maharajgunj Medical Campus, Tribhuvan University Kathmandu Nepal; ^2^ Sustainable Study and Research Institute Kathmandu Nepal; ^3^ Centre for Health Economics University of York York UK; ^4^ Active Pharmacy Pvt. Ltd. Kathmandu Nepal

**Keywords:** COVID‐19, case fatality rate, Nepal

## Abstract

The first COVID‐19 case in Nepal was reported on January 23, 2020. Then infection, then, started to spread gradually, and October marked the most devastating increase in COVID‐19 cases of the year 2020. Compared with the October 2020 peak in Nepal, the May 2021 peak of COVID‐19 observed 2‐ and 10‐fold rise in new cases and deaths per day, respectively. Given that this surprising increase in the death rate was not observed in other countries, this study analyzed the COVID‐19 case fatality rates between the two peaks in Nepal. We found an increase in death rates among younger adults and people without comorbidities.

## 1 INTRODUCTION

**FIGURE 1:**
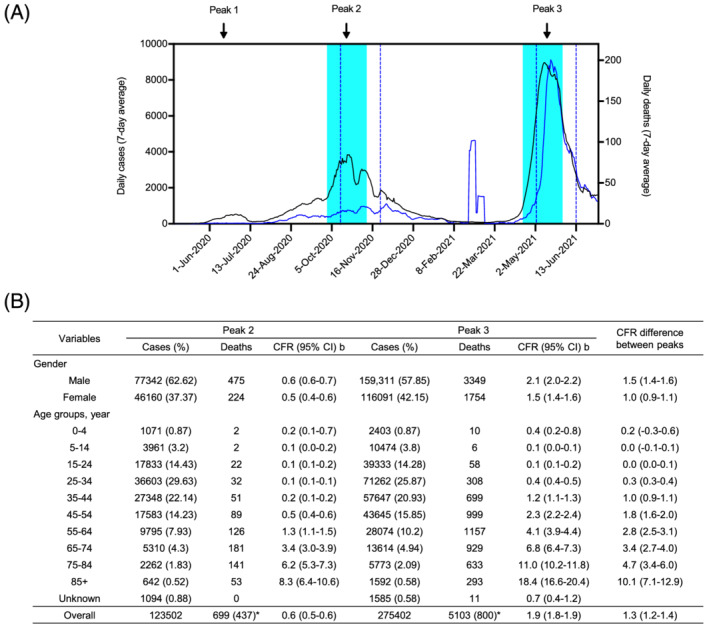
Situation analysis of COVID‐19 case fatality rate in Nepal. (A) Seven‐day moving average of the daily COVID‐19 infections (black) and deaths (blue). The number of days of second and third peaks used for the analysis in this manuscript are shown by cyan fill and blue lines for cases and deaths, respectively. (B) Confirmed cases and deaths attributed to COVID‐19 during two independent 6‐week durations highlighted in Figure (A). Data are taken from the official situation reports of the Ministry of Health and Population, Nepal, and WHO Nepal Situation Report on COVID‐19. ^b^Case fatality rate during a specific period was calculated as the total number of deaths/total number of cases during the same time frame preceding 2 weeks. An * indicates death with any known comorbidity

Nepal reported its first COVID‐19 case on January 23, 2020, within 2 months of the global index case identified in China. Two additional months were required for the second case to be diagnosed.^1^ Immediately after the second case, Nepal started to take stern measures to tackle COVID‐19. One of the most crucial management strategies Nepal took was imposing the national level lockdowns, which were relaxed and strengthened as the number of new cases reduced or started to rise again, respectively, evident from three incidences of an increase in COVID‐19 infection during the span of a year (Figure 1A). Early epidemiological analysis of deaths attributed to COVID‐19 showed that, as opposed to many countries, Nepal had a relatively lower case fatality rate (CFR) of 0.34%. In addition, females were more likely to die from the disease than their male counterparts.^2^ An understanding of the disease situation at the national level, followed by identifying high‐risk populations for SARS‐CoV‐2 infection and fatality, holds significant implications for developing and executing progressive public health surveillance and mitigation actions in Nepal. This research aimed to analyze COVID‐19 infection progression during the two most recent infection peaks observed in Nepal.

## 2 MATERIALS AND METHODS

We used the epidemiological data published by the Ministry of Health and Population (MoHP), Nepal,^3^ and the World Health Organization^4^ to calculate confirmed cases and fatality rates and compare the public health impact of COVID‐19. To calculate the differences of CFR between two different peaks from additional countries that displayed peak patterns similar to that of Nepal, we downloaded the data from a public repository maintained at ourworldindata.^5^ The numerator used to calculate CFR was the total number of fatalities with 14‐day lag time.^6^ To perform the comparative analysis of the spike protein, we used the genome sequences of the Nepalese SARS‐CoV‐2 strains and the Wuhan‐1 strain available in National Center for Biotechnology Information (NCBI).

## 3 RESULTS

The analysis of COVID‐19 infection and death showed that Nepal had three independent peaks of infection. The most recent peak occurred during April to June 2021, overwhelmed health care institutions and was the most devastating spell, with average infections and deaths accounting for 9000 and 200 per day, respectively (Figure 1A). This report analyzed the cases and deaths from the 6‐week time frames of Peaks 2 and 3. When we compared the number of cases and deaths between the most recent and preceding peaks, we found that the number of deaths increased by 10‐fold. In contrast, number of cases increased by about less than 3‐fold, indicating a significant increase in the CFR. Next, we aimed to identify the most vulnerable population during the most recent peak. Therefore, we analyzed all the deaths in terms of age, sex, and comorbidities. A total of 123,502 cases, 699 deaths, and 275,402 cases, 5103 deaths, were reported during the second and third peaks, respectively. Although the number of fatalities with known comorbidities relatively decreased, suggesting a significant rise in fatality among the healthy population, the detailed analysis of comorbid conditions and their link to death was impossible due to the lack of data (Figure 1B). While there was not a big difference between sex‐specific and age‐specific morbidities within the peaks, in Peak 3, the male had relatively higher mortality. Besides, the CFR had also increased across most age strata, and we found a remarkable rise in CFR among the younger age groups of 35–64 years. This indicated that the younger population is still vulnerable to disease in Nepal.

**FIGURE 2:**
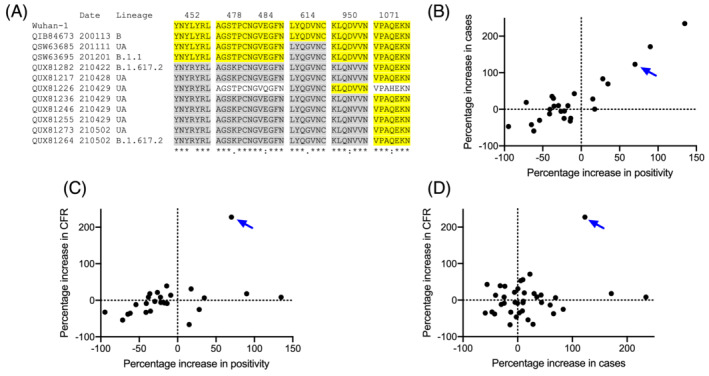
Evolutionary analysis and causal relationship of case fatality rate (CFR) in Nepal. (A) NCBI accession number, sample collection date, lineage, and sequence alignment of the 11 spike proteins from Nepalese SARS‐CoV‐2 strains, with publicly available genome sequence and comparison with the Wuhan‐1 strain. UA, unassigned lineage. (B) Relationship between a change in positivity rate and the number of cases. (C) Relationship between a change in positivity and CFR. Data include 25 countries that reported test positivity results and had two recent infection peaks similar to Nepal (B, C). (D) Relationship between a change in the number of cases and CFR among 38 countries with two recent infection peaks similar to Nepal. A blue arrow indicates Nepal's position in the figures (B–D), and the detailed dataset is available as the supporting information dataset

Genetic variants of SARS‐CoV‐2, through mutation, have occurred over time. To identify the link between the occurrence of SARS‐CoV‐2 variants and an increase in the number of incidence and deaths in Nepal, we first analyzed the publicly available genome sequence data for genomic surveillance. Although Nepal lacks in the field of genomic epidemiology, researchers have attempted to sequence and perform genomic epidemiological analysis of the SARS‐CoV‐2 genome identified in Nepal.^7^ Besides, the government of Nepal has also received significant support from the World Health Organization in this issue.^3^ Compared with the Wuhan‐1 strain, at least six mutations in the spike protein were identified in Nepal (Figure 2A). Furthermore, the genomic analysis performed during the second quarter of 2021 showed the presence of alpha (B.1.1.7), kappa (B.1.617.1), and delta (B.1.617.2) variants in Nepal. Among them, delta variant was the most prevalent, with an overall prevalence of more than 95%.^3^ Given that the alpha variant was first identified in the United Kingdom much earlier than the kappa and delta variants were identified in India,^8^ the predominance of delta variant in Nepal is also correlated with the higher transmissibility. Furthermore, nearly 20% of the delta variants spreading across Nepal had an additional K417N mutation in the spike protein.

To examine the link between the SARS‐CoV‐2 variants prevalent in Nepal and the death rates, we first identified and analyzed the reports from the countries that displayed a similar two‐phase peak of COVID‐19 infection. We found that an increase in positivity ratio was a factor responsible for the rise in number of cases between the peaks and the situation in Nepal was not an uncommon one (Figure 2B). Next, we analyzed the CFR between the two peaks of these nations and found that Nepal had the highest (227%) increase in the CFR between the peaks. With the most predominant delta variant, India reported an increase of more than 200% cases, but only about 10% increase in CFR during the most recent peak. This indicated that the delta variant alone may not be responsible for the increase in deaths in Nepal. Next, we aimed to examine if the overwhelmed increase in infection cases was a factor and found that both the increase in positivity and the number of cases did not explain the rise in CFR in Nepal (Figure 2C,D). These results indicated that some unknown factors might have played a role in the increase.

## 4 DISCUSSION

In summary, we performed a comparative analysis of the COVID‐19 infections and deaths during the two most recent peaks in Nepal. We also compared the data from Nepal with the countries that recently displayed two similar peaks. Although Nepal had increased the number of daily tests, the increase in percentage positivity among tested individuals was the major factor in the increase in cases. This could be attributed to the relatively slower growth in the number of tests performed than the rise in the number of cases. When available, the COVID‐19 tests were limited to targeted testing of symptomatic patients and risk populations. We found that the delta variant was widespread during the most recent peak in Nepal. Although this variant has an increased transmission capacity, higher hospitalization, secondary attack rates, and increased infection rates in younger groups, its CFR was reported to be relatively lower^9^, ^10^; in Nepal, the CFR increased nearly three‐fold. Although this might be attributed to the poor health care system in Nepal, further studies are required to identify the reason more specifically. As Nepal's death rate remained lower during the early phase of infection,^2^ the recent rapid increase in fatality rate is of serious concern. Besides, there is a possibility of unidentified and unreported cases at the community level, potentially leading to new epicenters. Likewise, the slow rate of vaccine inoculation across regions also imperils the outbreak of future infections.

## AUTHOR CONTRIBUTIONS

**Sulochan GC:** Conceptualization; methodology. **Ashok Khanal:** Conceptualization; methodology. **Atmika Paudel:** Data curation; investigation; supervision; validation. **Vijay S. GC:** Data curation. **Aashis Khanal:** Software.

### PEER REVIEW

The peer review history for this article is available at https://publons.com/publon/10.1111/irv.12922.

## Supporting information


**Data S1** Supporting information

## Data Availability

The data that support the findings of this study (Figure 1) are available from the corresponding author upon reasonable request. The data that support the findings of this study (Figure 2B–D) are available in the supporting information of this article.
